# Patenting of University and Non-University Public Research Organisations in Germany: Evidence from Patent Applications for Medical Research Results

**DOI:** 10.1371/journal.pone.0014059

**Published:** 2010-11-18

**Authors:** Peter Tinnemann, Jonas Özbay, Victoria A. Saint, Stefan N. Willich

**Affiliations:** Institute for Social Medicine, Epidemiology and Health Economics, Charité University Medical Center, Berlin, Germany; Universidad Peruana Cayetano Heredia, Peru

## Abstract

**Background:**

Patents are one of the most important forms of intellectual property. They grant a time-limited exclusivity on the use of an invention allowing the recuperation of research costs. The use of patents is fiercely debated for medical innovation and especially controversial for publicly funded research, where the patent holder is an institution accountable to public interest. Despite this controversy, for the situation in Germany almost no empirical information exists. The purpose of this study is to examine the amount, types and trends of patent applications for health products submitted by German public research organisations.

**Methods/Principal Findings:**

We conducted a systematic search for patent documents using the publicly accessible database search interface of the German Patent and Trademark Office. We defined keywords and search criteria and developed search patterns for the database request. We retrieved documents with application date between 1988 and 2006 and processed the collected data stepwise to compile the most relevant documents in patent families for further analysis. We developed a rationale and present individual steps of a systematic method to request and process patent data from a publicly accessible database. We retrieved and processed 10194 patent documents. Out of these, we identified 1772 relevant patent families, applied for by 193 different universities and non-university public research organisations. 827 (47%) of these patent families contained granted patents. The number of patent applications submitted by universities and university-affiliated institutions more than tripled since the introduction of legal reforms in 2002, constituting almost half of all patent applications and accounting for most of the post-reform increase. Patenting of most non-university public research organisations remained stable.

**Conclusions:**

We search, process and analyse patent applications from publicly accessible databases. Internationally mounting evidence questions the viability of policies to increase commercial exploitation of publicly funded research results. To evaluate the outcome of research policies a transparent evidence base for public debate is needed in Germany.

## Introduction

Intellectual Property (IP) protection and its effects on health have been controversially discussed for years. On the one hand, IP protection is considered essential as an incentive for innovation and investment in research and development (R&D) [1], [2], [3]. Researching entities can expect to recoup the extensive costs of successful research since they are granted exclusive protection on production and marketing of a resulting commercial product. On the other hand IP protection is considered as conflicting with human rights [4], [5], especially where prices for pharmaceutical products are regarded as inhibiting access to health products [6]. Exclusivity of products, brought about through patenting or exclusive licencing, can affect pharmaceutical prices and affordability, especially in developing countries [7].

An important part of knowledge generation is financed by public funding [8], [9]. In particular, the initial stages of R&D are performed by universities and other publicly funded institutions [10]. Beginning in the US, there has been a consistent trend of increasing IP protection of publicly financed research [11], [12], [13], [14]. In the context of publicly funded research, IP protection is facing an even more complicated set of supporting and challenging arguments in addition to those mentioned above.

Arguments supporting IP protection of publicly funded research include:

1. Publicly funded research organisations have limited budgets – the licensing of IP rights is believed to bring additional funding for R&D [15].
2. Publicly funded basic research that can be commercialized could spur science-based economic growth [12], [16].
3. Implementing IP management might be necessary for bridging the technology transfer gap. Private investors for advanced stages of R&D can only be found if exclusivity by IP protection has already been obtained [17].

Arguments opposing IP protection of publicly funded research include:

1. IP protection could hamper the possibilities of further research, cutting off researchers from using the results of others [18], [19].
2. Many public research institutions in the US spend more on IP protection than they earn from it. In these cases, IP protection is not financially beneficial [20].
3. IP protection can lead to increased prices for products to patients and public health systems [21]
4. From a political and moral standpoint, the results of publicly funded research should always be public and not used for private benefit [18].

In Germany during the late 1980s and 1990s, global free-market forces led the German government to push for greater commercialisation of research. Firstly, society had begun to expect a return on investment from public research and, secondly, the realisation of the shift from an industrial to knowledge based society put emphasis on the exploitation of knowledge [16]. In 2001, the German Federal Ministry of Education and Research initiated a research policy shift to push for utilisation (*Verwertungsoffensive*) of research results [22]. Within this policy shift the German framework for IP on university research was changed with the 2002 reform of the Law on Employees' Inventions (*Gesetz über Arbeitnehmererfindungen*) [23].

These adjustments were modelled on the results of the fundamental changes in public patenting brought about by the Bayh-Dole Act in the US in 1980 [24]. In Germany before 2002, university professors had the freedom to decide whether or not they wanted to patent inventions resulting from their work. They were invited to use the services of university technology transfer offices (TTOs). The 2002 legal reform altered the situation: All professional inventions became attributed to the university. The university is entitled to claim the invention from the inventor and to apply for a patent. Only if the university decides not to exert this right, are professors or researchers allowed to file a patent application by themselves. Publicly funded research organisations in Germany are still not, however, required by law to disclose any patents on inventions arising from their research.

Although the issue is controversially debated internationally, little empirical data is available concerning IP on health products from publicly funded research. Some data on the situation in the US have been analysed [12], [25], [26]. In Germany, until now only a general analysis of university patents has been performed [27]. Data on some patent applications from German medical faculties is available online [28], but the detailed methodology used to collect this data has not been published. A long-term evaluation of patenting of health products by universities and non-university public research organisations (PRO) in Germany has not been conducted. To enable an informed debate, the objective of this study is to analyse how patents on health products, applied for by universities and non-university PROs in Germany, can be identified and how many patents these institutions have applied for.

## Methods

We decided to take on a patent research approach. We extracted patent documents concerning health products developed by German universities and non-university PROs from the publicly accessible database of the European Patent Office (EPO). We used the DEPATISnet advanced search interface provided by the German Patent and Trademark Office (DPMA, Deutsches Patent- und Markenamt) [29].

We defined our search criteria as:

1. Patent documents returned by a search request on October 10, 2008, using the DPMA online search interface
2. Documents referring to patent applications submitted between January 1, 1988 and December 31, 2006
3. Applications originating from an applicant in Germany
4. Applicants being either a
  - university or university-affiliated institution or a
  - non-university PRO, i.e. a member of Helmholtz-Gemeinschaft, Max-Planck-Gesellschaft, Leibniz-Gemeinschaft or Fraunhofer-Gesellschaft or a
  - “Patentverwertungsagentur” (equivalent to a TTO) of universities or non-university PROs, i.e. a member organisation of TechnologieAllianz [30] or a
  - federal institution of the German state
5. Documents relating to a product classified in the sector of “medical science” according to the International Patent Classification (IPC) main class [31].

The four main non-university PROs were considered. The Helmholtz Gemeinschaft (Helmholtz Association) is a community of scientific-technical and biological-medical research centres commissioned to pursue long-term research goals on behalf of the state and society [32]. The Max-Planck-Gesellschaft (Max Planck Society) conducts basic research in life sciences in the interest of the general public. The Society takes up research areas to complement work done at universities or that universities are not in a position to accommodate or deal with adequately [33]. The Leibniz Gemeinschaft (Leibniz Association) research institutions address scientific issues of importance to society as a whole. They conduct natural science research cooperating with universities and complementing university research while their academic staff also hold joint academic appointments [34]. The Fraunhofer-Gesellschaft is Europe's largest application-oriented research organization, promoting and undertaking applied research for direct utility to private and public enterprise and of wide benefit to society as a whole. The Society aims to promote the economic development with particular regard for social welfare and environmental compatibility [35].

We designed our search request to increase sensitivity despite the high number of spelling (and mis-spelling) variants in applicant names.

The IPC classifies patents by their main purpose. We searched for several subclasses of IPC class A61 (“Medical or veterinary science; Hygiene”), excluding all subclasses with other purposes than human medicine. Patent applications only classified in IPC section C (“Chemistry; Metallurgy”) were not searched for, as within this section it is not possible to distinguish between inventions for medical purposes and others inventions. Discussion of ‘patents’ hereafter refers only to patents in the above IPC classification. For a detailed description on the search request used, refer to Annex S1.

Inventions originating from research at universities are not included if patents were solely filed by industry research partners.

We subsequently processed the resulting datasets through a series of steps. In further steps, we added extra columns for data calculated from pre-existing columns. (Explanations of the rationale for introducing each step and the resulting number of datasets after each step are presented in the Results section.)

### Initial data processing

**Step** **Task**

1) We assembled data gathered by database requests to the DPMA interface in a Microsoft Access 2003 database.
2) We deleted duplicate entries from the database.
3) We deleted documents on utility models.

### Applicant data processing

**Step** **Task**

4) We extracted the content of the “applicant” column into a separate applicant table.
5) From this applicant table, we removed applicant entries where no applicant met our inclusion criteria.
6) For the remaining entries in the applicant table, we developed the table into a substitution list: We associated each original applicant entry with up to five single applicants in a consistent notation.
7) We associated each applicant with one of the following groups:
  - University or university-affiliated institution
  - Fraunhofer-Gesellschaft
  - Helmholtz-Gemeinschaft
  - Leibniz-Gemeinschaft
  - Max-Planck-Gesellschaft
  - TTO (Patentverwertungsagentur)
  - Federal institution
  - others
  - hide from table (applicants not covered by inclusion criteria)
8) We associated the main table from step 3 (see above) with the applicant substitution table from step 4–7.

### Patent family processing

Many patent applications are first filed in the applicant's home country and only later submitted in other countries, at the European Patent Office (EPO) or at the World Intellectual Property Organization (WIPO). This results in so-called “patent families”, constituted by several patent documents – often from different countries – all referring to a single first patent application based on the same research result (invention). As we relied on the DPMA search engine's patent family search, we used the DPMA definition of a patent family as “a group of patent applications and grants […] which are all directly or indirectly related to each other by way of a common priority” [36].

We considered only the most relevant document from each patent family and excluded additional documents within the same patent family. Deciding on the most relevant document in a patent family, however, is not a straightforward task. For our purposes, our first decision criterion was the scope of a patent document: If a patent had been submitted to WIPO, this document was considered the most relevant. Second highest relevance was given to documents from applications to EPO. Only if there were no documents from applications to WIPO or EPO, was the first application in a single country considered as the most relevant document. If there were several documents with equal scope in a patent family, we considered the document with the earliest application date to be the most relevant.

**Step** **Task**

9) Using a patent family request (for details see Annex S2), we evaluated the patent family for each document: If one patent application inside a patent family had been granted, we tagged all documents in this family as granted. In the following steps, we used only the most relevant document in each family. We hid all other documents from the table.

### Data preparation for statistical evaluation

**Step** **Task**

10) We created a new table containing one entry per document per applicant using a UNION-request (for details see Annex S3). In this table a document with for example two applicants appears twice.
11) We created a new table only containing documents where at least one applicant is a university or university-affiliated institution using a selective request based on the data from step 9 (for details see Annex S3).
12) For the data from step 11 (see above), we created a new table containing one entry per document per applicant (similar to step 10 above).

### Further Data Processing

We imported the tables from steps 9 to 12 (see above) into Microsoft Excel 2003 worksheets. In each worksheet we added one column “application year”, based on the already available application date information. Additionally, we added one column “country code”, based on the country information available from the document identification number.

We added another column, grouping country codes into one of the following groups:

- International patent application – according to Patent Cooperation Treaty (PCT)
- European patent application
- Patent application in Germany
- Patent application in other countries

We added a column “weighting”. Its content was calculated as one divided by the number of applicants.

Only patent application documents submitted between 1 January 1988 and 31 December 2006 were included, given that we conducted our first database search on 1 July 2008 and 18 months before this search date no public disclosure of application submission is required.

We processed all retrieved patent applications that matched our search criteria. Because of possible bias due to the different patenting policy in eastern Germany (GDR) until 1990, we also performed subgroup analysis for the time period from 1997 to 2006 to analyse accessible applications of the past 10 years.

Given the high number of documents to be considered, an exhaustive case-by-case evaluation to identify patent families was not feasible. We therefore used a step-wise approach: If a patent family contained an international (WIPO) application under the PCT, we considered this document as the most relevant. An international application theoretically enables the applicant to request a patent in each of the 141 countries signatory to the PCT [3]. When there was no international application, we looked for an EPO application, valid for 35 member states as of January 2009 [37]. If no multi-country (WIPO or EPO) application existed, we considered the first application in a single country as the most relevant. As we investigated R&D results from Germany only, this was also the country of the first application in almost all cases.

## Results

### Results of Data Processing

**Figure 1:**
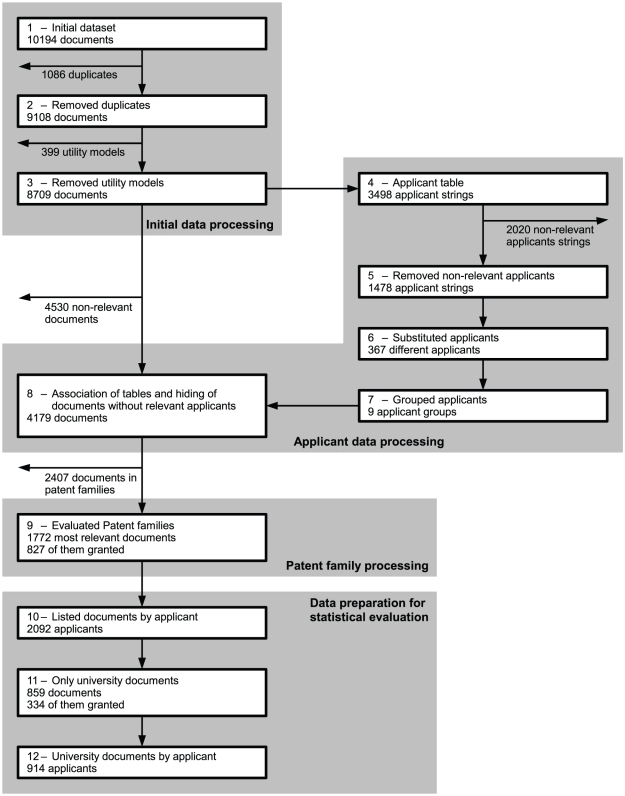
Stepwise Presentation of Results from Data Extraction and Processing.

Figure 1 presents a graphical depiction of the processing (by each numbered step) and results of data extraction.

The initial dataset retrieved using the DPMA search engine resulted in a total of 10194 entries (step 1). After deleting duplicate entries and documents referring to utility modes, 8709 documents remained (step 2 to step 3).

We then processed the applicant strings, removed documents where no applicant met our inclusion criteria and adjusted for variations in applicant spelling (step 4 to step 6). This resulted in 367 different applicants, which were stratified into nine groups (step 7). Of all 367 applicants, 193 matched our inclusion criteria. All others were excluded from further evaluation.

We associated the document datasets from step 3 with the applicant substitution table from step 7. We excluded 4530 patent documents (52.0%) for which applicants did not meet the inclusion criteria. The resulting database contained 4179 patent documents (step 8).

After evaluation of patent families (for details see Annex S2), 1772 documents were considered most relevant for their respective patent family. Out of these 1772 patent family documents, we marked 827 (46.7%) documents as granted in at least one country (step 9). The remaining 945 (53.3%) applications were either not granted or were still being processed.

When documents were listed per applicant, out of the 1772 documents mentioned above, 2092 applicants were considered. This resulted in an average of 1.18 applicants per document (step 10).

Considering only documents where at least one applicant was a university or university-affiliated institution, 859 documents remained (step 11). Of these, 334 (38.9%) had granted patents in their patent family. These documents had 914 applicants, an average of 1.06 applicants per document (step 12).

### Results of Dataset Analysis

**Figure 2:**
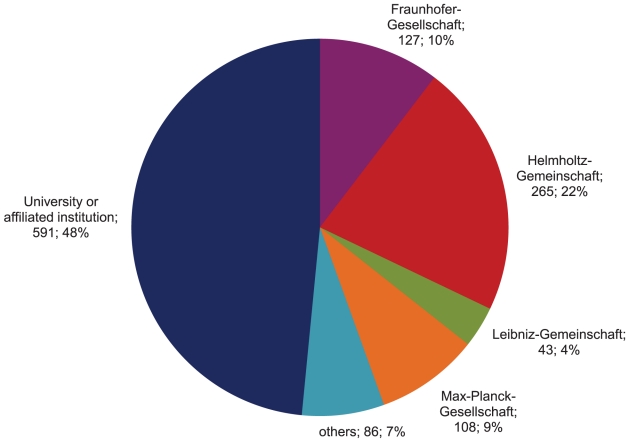
Number and percentage of medical-science patent applications (N = 1220) by German public research organisations, 1997–2006.

The distribution of patent applications on health products submitted by various German PROs in 10 years is shown in Figure 2. Universities and university-affiliated institutions submitted almost half (48%) of all PRO patent applications. In terms of the main non-university PROs, the institutes of Helmholtz-Gemeinschaft submitted one fifth of all patent applications on health products – more than double the number of applications from each of the other three non-university PROs individually (22% compared with 4–10%).

Technology transfer offices were hardly ever mentioned as an applicant. In some cases, the applicant was a federal agency of the Federal Republic of Germany.

**Figure 3:**
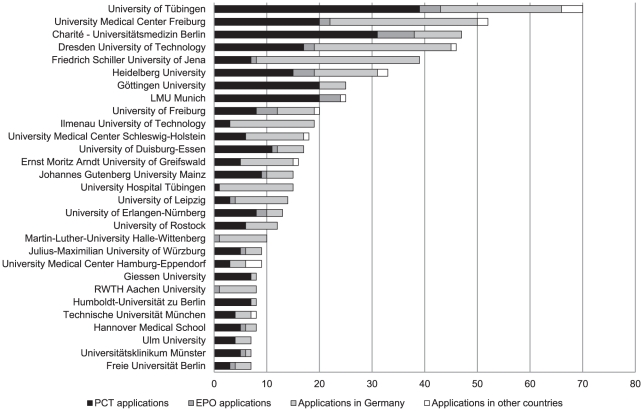
Number of medical-science patent applications submitted by German universities and university-affiliated institutions, 1997–2006.

The top German universities and university-affiliated institutions in terms of the number of patent applications submitted during the period 1997 to 2006 is shown in Figure 3. In some cases university-affiliated institutions appear independently from the university. For example, Charité – Universitätsmedizin Berlin appears independently of Humboldt University, and the University Hospital Freiburg appears separately from University Freiburg.

Over this ten-year period, no more than 70 patent applications on health products were submitted by any one university or university-affiliated institution. On average, 22 patent applications were submitted per university or university-affiliated institution during this period, and only the top eight were above this average – accounting for 60% of all applications.

**Figure 4:**
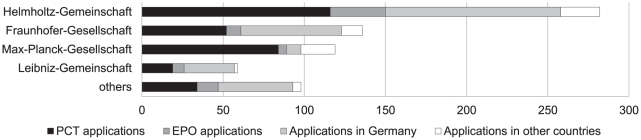
Number of medical-science patent applications submitted by main German non-university public research organisations, 1997–2006.

Figure 4 shows the patent applications for non-university PROs over the same ten-year period.

**Figure 5:**
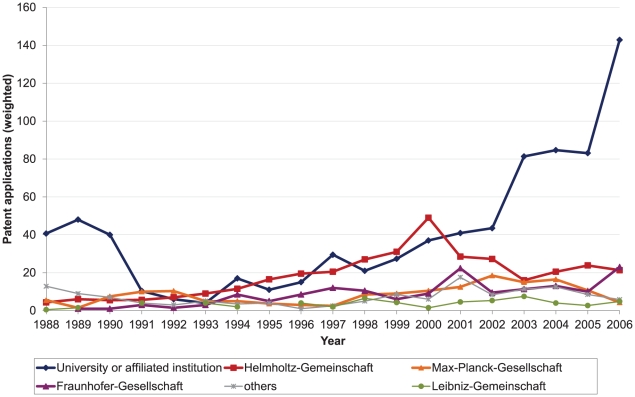
Number of medical-science patent applications per year by German public research organisations, 1988–2006.

The number of patent applications by all PRO groups per year for the period 1988 to 2006 is shown in Figure 5. The German IP legal reforms were introduced in 2002. The number of patent applications per year submitted by university and university-affiliated institutions has more than tripled since then, from 44 in 2002 to 143 in 2006. The number of applications by the other non-university PROs, however, has remained relatively stable before and since 2002.

The number of patent applications submitted by universities and Helmholtz-Gemeinschaft also doubled between the mid-1990s and the early 21^st^ century – before the reforms. Since 2000, however, the number of patent applications submitted by the Helmholtz-Gemeinschaft has decreased by more than half. German reunification in 1990 changed patenting policy. A significant decrease in patent applications by university and associated institutions from 1989 to the early 1990s can be noted. Finally, medical-science research by university and university-affiliated institutions still amounts to less than 150 PCT applications for health products in 2006 - and much less before that.

**Figure 6:**
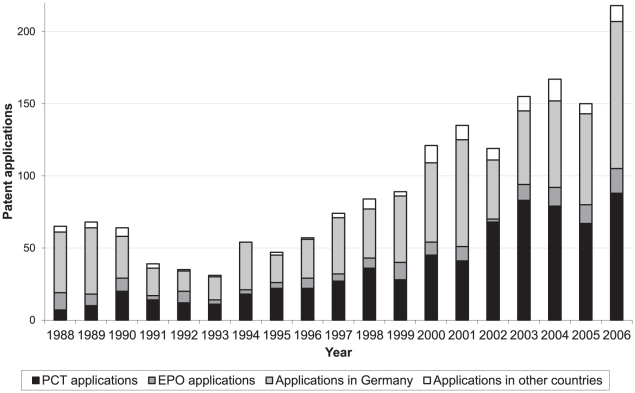
Number of patent applications by German public research organisations submitted at different patent offices, 1988–2006.

Figure 6 shows the distribution of the highest level patent applications submitted to each type of patent office. More than 40% of all patent applications were only submitted to the German Patent and Trademark Office (DPMA) and approximately 40% were submitted to WIPO for the PCT process in addition to the first application at some national patent office. Less than 10% of all patents were applied for at the European level (EPO), but not submitted to WIPO for the PCT process, and even less were only submitted to a foreign patent office, for example the US Patent and Trademark Office (USPTO).

Over the years, applications to only DPMA and EPO have relatively decreased, while the percentage of PCT applications has increased relatively and absolutely.

**Figure 7:**
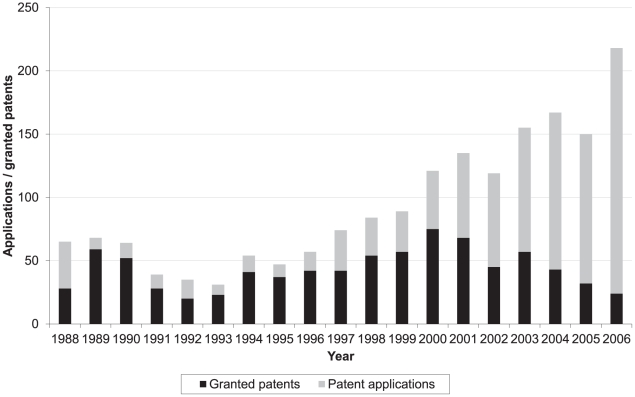
Patent applications and granted patents by year of application, 1988–2006.

In the final stage of the analysis, we examined the number of patent applications and patent grants per year by German PROs for the period 1988 to 2006 (Figure 7). The total number of patent applications submitted annually increased quite steadily during this period: from around 35 applications per year in the early 1990s to 218 applications in 2006. The proportion of granted patents among these applications is on average 70% until 2000, decreasing since then.

The data provided by our source does not allow to distinguish between denied and pending applications. Therefore the closer we get to the present day, the higher the proportion of patent applications from recent years which are still pending.

## Discussion

German government policy on patenting aims to create more opportunities to exploit research results commercially, in particular those from publicly funded research. The debate on publicly funded research and its associated responsibility towards the public is gaining momentum. The debate originated around commercialisation of university inventions and then gathered increasing attention in relation to cases of patents and licences for medicines, with specific focus on access to them in the developing world [26], [38], [39]. The issue, however, is becoming increasingly debated in high-income countries as well. Health systems financing, its relevant associated governmental policies, and the rising costs for pharmaceutical products are coming increasingly into the focus of public attention [40], [41].

The purpose of this research is to examine the patterns of patenting on health products, applied for by German university and non-university PROs. This research will enable and contribute to an informed debate on patenting by PROs in Germany.

In Germany, the patent applicant is not required by law to disclose information on patents resulting from publicly funded research. The DPMA is legally required after 18 months to publish details for all patents applied for. Therefore, the DPMA database is the only publicly accessible data source that comprehensively details all patents applied for in Germany.

Since we could not identify any established methods to answer our questions, we developed a new method to search for and process data from patent applications. We chose to acquire our patent data through the DPMA database to allow for systematic analysis and to avoid various biases associated with alternative methods, such as surveying research institutions on patenting.

We begin with a discussion of this method before moving on to discussion of our research findings.

### Discussion of Method

We developed and present a new method to extract and process data on patent applications for medical science inventions by universities and PROs in Germany using a publicly accessible database. Using our method we were able to identify and retrieve patent documents selectively for a defined group of applicants and covering a defined field of inventions. We searched the DPMA database for patent documents from universities and non-university PROs in the field of health products. We extracted necessary data from identified documents and processed them for further statistical analysis of applicant activity, range of patent protection, IPC main class or longitudinal development.

A search request sent to the DPMA advanced search interface can combine several search criteria, however searches soon become highly complex. Careful attention must be paid to drafting the search request in a way that corrects for the high number of variations in spelling and typographic errors in the database. For example, amongst applicants we found eleven different versions of spelling for “Charité Universitätsmedizin Berlin”, the medical faculty of the Berlin universities. Labour-intensive processing is necessary to exclude false-positives (52% of all documents), harmonize all spellings and to prepare data for further analysis.

The classification of patent application documents is also not entirely consistent across patent documents from within one patent family. As we only included documents classified by patent offices as health products according to IPC, we may have missed other relevant inventions. For example, inventions classified in chemistry or cosmetics may later be used for medical purposes. Publicly funded research in a medical research laboratory may also result in an invention that is relevant for and patented under another category, for example, information technology.

Documents were flagged as “granted” if at least one document in their patent family was a document granting a patent. We used this simplification to allow for quantitative analysis. WIPO does not grant patents; it only facilitates the application process. Therefore, WIPO applications (identified as the most relevant document in the patent family) were flagged as granted if the patent family contained, for example, a granted patent from EPO. In many cases, however, other countries simply follow the grant decision of EPO as they cannot afford the same level of extensive search and patentability checking. The time lag between application and granting of a patent also requires such simplification, as applications granted by one patent office may still be pending by another.

The complexity and number of issues we encountered in developing this method highlights how complex the patent database system is to use. The resulting datasets require several steps of refinement before being suitable for statistical analysis. Nevertheless, we believe our stepwise method can be replicated by others for different IPC classes in Germany. In term of generalisability, we believe our stepwise approach could be applied to similar databases available in other countries.

### Increased patenting by universities and university-affiliated institutions

Since the 1990s there has been a clear and substantial increase in the number of patent applications submitted each year by German PROs. German university and university-affiliated institutions have submitted an increasing number of patents applications and are responsible for most of the overall increase. This has largely taken place since the 2002 legislative reforms. Other non-university PROs, however, were not found to be submitting patent applications at a higher rate, and there are even indications of a trend of decreased patent submissions.

We consider that a significant part of the increase may be because patent applications previously submitted by individual researchers are now registered under the university name. This would be in accordance with the 2002 legal reforms in Germany. Other researchers have found clear empirical evidence from European countries, including Germany, that the number of university-invented patents is much higher than the number of patents owned by universities [42]. This might be related to the tendency in the 1980s and 1990s for patent ownership to be assigned to the industry partner that financed the research project, with researchers and professors only included in the list of inventors.

Very few assessments have been conducted analysing the number of patents applied for by individual researchers or research organisations in Germany prior to the 2002 legislation changes [43], [44]. It therefore remains questionable whether research at university and university-affiliated institutions has resulted in more patents and commercially developed products. In fact, it appears to the authors nearly impossible methodologically to collect such figures from existing patent databases, since this would require searching for individual researchers. Furthermore, the databases do not indicate if patented inventions are a result of publicly funded research.

Patent applications by non-university PROs other than Helmholtz-Gemeinschaft have remained relatively stable over time and do not show the post-reform increases demonstrated by universities. The number of patent applications submitted by universities and Helmholtz-Gemeinschaft doubled between the mid-1990s and early 2000s, before the legislation reforms were introduced [16], [22]. This points to the importance of factors other than national legal change.

So *et al* warns against interpreting data that patents have increased as evidence of increased commercialisation or actual technology transfer of public research [14]. Furthermore, they caution against attributing such increases to changes in national IP policies. There is some evidence that the international rise in patenting by universities has been driven by increases in technological opportunities in the biomedical and biotechnology sectors, more so than by IP policy changes [43], [45]. Alternatively, researchers, TTOs and universities may feel a great pressure (real or perceived) to submit more patent applications, as patenting becomes increasingly expected and a common evaluative indicator for individual research output performance.

Our finding of an increase in patent applications could be an indication that the legal reforms have been successfully applied with the instigation of the TTOs (Patentverwertungsagenturen). A study by Saragossi and Van Pottelsberghe found that the increase in patents in Belgian universities was also related to more effective TTOs [46], although a simple increase in TTO quantity could also be responsible for the increase in patenting. In line with this, Baldini found that in Italy a lack of university support mechanisms – including TTOs and a lack of funds to cover patenting costs – were associated with lower patenting, while availability of administrative and TTO structures was associated with increased patenting [47], [48].

The number of patent applications by university institutions decreased significantly from 1989 to the early 1990s. The decrease appears to be a reduction predominantly in East German patent applications. We believe this reflects different patent legislation and patenting policies at universities in the former German Democratic Republic.

### Time-lag between patent submissions and outcomes

The increase in patent applications during the 1990s was accompanied by an increase in the number of applications granted. From 2001, however, patent applications continued to increase while the number and proportion of granted patents found in the database decreased. The data provided by the patent database does not allow for distinguishing between rejected and still pending applications. According to German patent law (patG, §31 [2]2.), while patent offices are required to publish applications after 18 months, issuing a decision on whether or not the patent will be granted takes on average around 2–2.5 years [49].

This could explain why only 46.7% of the documents in our list are marked as granted, whereas annual data on applications with completed examination suggests a grant rate in Germany of about 53% [50]. We can therefore assume that about 6% of total applications are still pending, mainly those filed in the most recent years.

### Heterogeneity of universities and university-affiliated institutions

We calculated the top 25 German universities and university-affiliated institutions in terms of the number of patent applications submitted between 1997 and 2006. The number of applications submitted by each entity was relatively low – on average 22 applications per university and university-affiliated institute, with the top eight universities accounting for 60% of all applications.

Baldini showed that the availability of a medical school to the university was related to higher patenting activity at Italian universities [47], [48]. In our analysis, amongst the top 25 patent submitting universities in Germany, only the University of Ilmenau had no medical faculty, although they have an Institute for Biomedical Engineering and Informatics.

We could not conclusively determine a specific communality between the universities with the highest patenting activity, such as university size or size of the city where the university is located. Thus, for the most part our findings are in line with the evidence that other factors play a significant role in the patenting activity of individual universities. Such factors include the financial resources and level of R&D funding, the economy and industrial strength and structure in the university's surroundings, royalty distribution practices and the university's commitment to exploit inventions [45], [47]. Thus, it cannot simply be assumed that universities are the most productive research institutions or that others, such as Helmholtz-Gemeinschaft, are more efficient than its other non-university PRO counterparts.

The relatively small number of patent applications submitted is striking. All medical-science research from universities and university-affiliated institutions still amounted to less than 150 WIPO patents applications in 2006 and much less before that. A study by Malik revealed that US and UK universities patent much more than Germany or France [51]. For the period 1994 to 2005, the US submitted 18.9 biotechnology patents per million inhabitants, while Germany submitted only 0.11 patents per million inhabitants. Lehrer discusses university entrepreneurialism, postulating that it depends on ‘structured interface between the invisible hand of market forces and the visible hand of public R&D funding’ [52].

### Policies and responsibilities for patenting of publicly funded research

The policy reforms on patenting by universities and non-university PROs in Germany were designed to increase the technology transfer and commercialisation of publicly funded research. Increases in patent submissions or approved patents do not necessarily indicate increased or improved commercialisation. Patent applications are not of equal value – the commercial potential of patents differs greatly and may change over time. Also, the commercial viability or successful application of a patented invention is not always guaranteed. Econometric evidence from the US indicates that revenue from increased commercialisation of public research does not necessarily outweigh costs associated with patent management [14]. If the same pattern applies for Germany, increased patenting may hamper economic and knowledge development.

The evidence from the US and other countries draws into question the underlying objective of the German policy changes. From patenting figures presented here, it is not possible to look into the commercial success or sustainability of the increased patenting. To our knowledge, in Germany figures on commercial returns are neither collected systematically at an aggregated federal level or made publicly available at patent or institutional level. The justification of these policies and their evaluation needs to be rethought and indicators should be developed that provide for an evidence based evaluation. Further, we believe that, in general, more transparency is needed regarding public funding of research and its socially meaningful outcomes. Therefore, in Germany and elsewhere, by law public research funding should be linked to its measurable outputs, not only in the form of patents, but also in terms of commercialisation of these patents.

At a more complex level is a consideration of what the value and consequences of a patented invention implies. So *et al* emphasise that the benefits of patenting and licensing must always be weighted against the social cost of a licenses' exclusivity [14]. The commercial and the societal value of a particular invention may be very different. Medical research on tropical diseases is often neglected because its profitable commercial value is low - and yet its social value may be very high, potentially saving millions of lives or life-years. The commercial and societal value of different inventions and patents can vary depending on what ‘society’ is considered. The value of a patent for a drug needed by the poor in a low-income country will be different than when considering its worldwide implications or applications. Finally, the social value is very difficult and controversial to define.

We found that the majority of the increase in patent applications has been for submissions either to WIPO or only to the German Patent and Trademark Office. A WIPO application might be only used in certain countries, for example only high-income countries such as the US and European states. But it can also lead to patents in almost all states of the world, including low-income countries. It could be the case that those applying are aiming for high-income countries with clear intentions for commercialisation. This would be in line with the theoretical underpinning and justifications of the legal reforms. We believe there is a need for more discussion, in Germany and internationally, on what responsibility PROs and national governments have to prevent or manage the negative consequences of increased patenting and licensing. What policies are or should be in place to clarify the responsibilities these institutions have to protect? Are these policies prioritising the public good and holding the institutions accountable to it? We believe in particular that policies on the use of publicly funded research must be transparent to the public.

Level of patenting has become an indicator for ranking the research output and effectiveness of universities. The traditional ranking in science is the number of publications, and indeed evidence suggests that number of publications and patenting levels are often associated [16], [52], [53]. However, the increase in patent applications may be a reflection of the effectiveness of the TTOs and administrative structures, rather than increased inventiveness or productivity of researchers at universities or non-university PROs. This seems to suggest that ranking patent submissions is a questionable indicator of the success, efficiency and productivity of a PRO. The use of patents as a ranking for inventiveness of PROs must therefore be reconsidered, or at least appropriately developed, in relation to publishing indexes and other established or verifiable indicators.

### Limitations

The complexity and number of issues we encountered in developing this method highlights how complex the use of a patent database system is. Though publicly available, mining empiric information from such a database is not a simple task; in fact it appears that the databases are designed for searching information on single patents. We are aware that the limited sensitivity of our method is not suitable for exhaustive evaluation of the patent landscape on a specific topic. Nonetheless, we have developed a multi-step method that allows IP laypersons – like researchers in the sector of medicine – to familiarize themselves with patents in their field of research.

It was not within the scope of the current analysis to weight or contextualise individual PROs, for example by PRO size or volume of research funding received, or even distinguish the applicability of such factors in contributing to meaningful interpretation of our results. Our data gives no information on licensing of patents – this is typically provided by commercial data providers.

Nevertheless, analysing patent applications is a first step to lay the foundation for further evaluation of the commercialisation of patents, the main argument for IP protection of publicly funded research.

### Conclusions

In 2002, IP legal reforms designed to encourage commercialisation of publicly funded research were introduced in Germany.

We have developed and present a new method to extract and process data on patent applications for medical science inventions by universities and non-university public research organisations in Germany using a publicly accessible database.

Overall, we identified 1772 health related patent families applied for by 193 different universities and non-university PROs since 1988. 872 (47%) of these families included granted patents.

Patenting by universities and university-affiliated institutions account for the majority of submitted patent applications. Since the introduction of IP reforms patent applications by universities have tripled and relatively increased for PCT countries, whereas non-university PROs patent applications have remained stable. However, there can be various reasons other than instigated policy changes for this increase in PROs' patent applications.

Empirical evidence analysing Bayh-Dole-type policy changes in developed countries, particularly the US, do not conclusively demonstrate the commercial viability of increased patenting of publicly funded inventions. Publicly available data on patenting from publicly funded research institutions in Germany does not allow conclusions to be drawn regarding commercial viability of patents.

Licensing agreements that commercially exploit patented inventions from publicly funded research need to be made publicly accessible, to allow for empirical, evidence based policy analysis.

In the future, publicly funded research results should not only be measured by their patenting and commercial successes, but also in terms of their social and global health relevance.

## Supporting Information

Annex S1 DPMA Database requests.
(0.05 MB DOC)

Annex S2 Patent family requests.
(0.03 MB DOC)

Annex S3 SQL requests.
(0.04 MB DOC)
