# Supplementary material for: Patenting of University and Non-University Public Research Organisations in Germany: Evidence from Patent Applications for Medical Research Results
Source: PLoS One. 2010 Nov 18;5(11):e14059. doi: 10.1371/journal.pone.0014059 (PMC2987808; doi:10.1371/journal.pone.0014059)
Supplement: Annex S1 — DPMA Database requests. (0.05 MB DOC) [file pone.0014059.s001.doc]

# Annex S1 - DPMA Database requests

## Main request

Based on the inclusion criteria explained in the methods section, search criteria were grouped in four columns:

| **A. Year** | **B. Class of application according to international patent classification (IPC)** | **C. Country of applicant** | **D. Applicant** |
| --- | --- | --- | --- |
| - 1988 - 1989   …   - 2006 | - Diagnosis; Surgery; Identification   - *excluding subclass* Identification of persons, e.g. finger-printing, foot-printing, impression techniques - Dentistry; oral or dental hygiene   - *excluding* *subclass* Devices for cleaning between the teeth - Filters implantable into blood vessels; Prostheses; Devices providing patency to, or preventing collapsing of, tubular structures of the body, e.g. stents; Orthopaedic, nursing or contraceptive devices; Fomentation; Treatment or protection of eyes or ears; Bandages, dressings or absorbent pads; First-aid kits - Artificial respiration or heart stimulation - Containers specially adapted for medical or pharmaceutical purposes - Devices or methods specially adapted for bringing pharmaceutical products into particular physical or administering forms - Devices for administering medicines orally, e.g. spoons; Pill counting devices; Arrangements for time indication or reminder for taking medicine - Preparations for medical, dental, or toilet purposes   - *excluding subclass* Cosmetics or similar toilet preparations - Methods or apparatus for sterilising materials or objects in general; Disinfection; Sterilisation, or deodorization of air; Chemical aspects of bandages, dressings, absorbent pads, or surgical articles   - *excluding* *subclass* Deodorant compositions - Devices for introducing media into, or onto, the body; Devices for transducing body media or for taking media from the body; Devices for producing or ending sleep or stupor - Electrotherapy; Magnetotherapy; Radiation therapy; Ultrasound therapy - Therapeutic activity of chemical compounds or medicinal preparations | - Federal Republic of Germany - German Democratic Republic | group 1   - Universities - Helmholtz community - Max Planck society - Leibniz community - Fraunhofer society - Borstel research centre   group 2   - Max Delbrück centre - German Cancer Research Centre - Berlin research association - Technology transfers offices of Tech­no­lo­gie­Allianz - Vakzine Projekt Management GmbH - other publicy financed institutes, research institutions and organizations |

The elements within each column were linked by a logical (Boolean operator) “OR”. Columns were linked by a logical “AND”. The search was performed serially in discrete requests for each year and for each of the two applicant groups D1 and D2 to stay below 500 results per search (hard limit of the search interface).

In total, 38 search requests were performed with the following search criteria

1. (1988) AND [B] AND [C] AND [D1]
2. (1988) AND [B] AND [C] AND [D2]
3. (1989) AND [B] AND [C] AND [D1]
4. … - 37. …
5. (2006) AND [B] AND [C] AND [D2]

The complete syntax of the search request was:

- for searches of type (year) AND [B] AND [C] AND [D1]:

*AY=(year) AND
((ICM=A61B? NOT ICM='A61B 5/117') OR (ICM=A61C? NOT ICM='A61C 15/?') OR ICM=A61F? OR ICM='A61H 31/?' OR ICM='A61J 1/?' OR ICM='A61J 3/?' OR ICM='A61J 7/?' OR (ICM=A61K? NOT ICM='A61K 8/?' NOT ICM='A61K 7/?') OR (ICM=A61L? NOT ICM='A61L 9/01?') OR ICM=A61M? OR ICM=A61N? OR ICM=A61P?) AND
(PA=DE OR PA=DD) AND
(PA=UNI? OR PA=HOCHS? OR PA=FAKULTAET? OR PA=HELMH? OR PA=PLANCK OR PA=LEIBNIZ OR PA=FRAUNH? OR PA=FORSCHUNGSZ? OR PA=BORSTaEL)*

- for searches of type (year) AND [B] AND [C] AND [D2]:

*AY=(year) AND
((ICM=A61B? NOT ICM='A61B 5/117') OR (ICM=A61C? NOT ICM='A61C 15/?') OR ICM=A61F? OR ICM='A61H 31/?' OR ICM='A61J 1/?' OR ICM='A61J 3/?' OR ICM='A61J 7/?' OR (ICM=A61K? NOT ICM='A61K 8/?' NOT ICM='A61K 7/?') OR (ICM=A61L? NOT ICM='A61L 9/01?') OR ICM=A61M? OR ICM=A61N? OR ICM=A61P?) AND
(PA=DE OR PA=DD) AND
(PA=FORSCHUNGSV? OR PA=DEUTSCH? OR PA=INST? OR PA=ZENTR? OR PA=CENTR? OR PA=VAKZ? OR PA=PATENTAL? OR PA=CAMPUS OR PA=ERFINDERZENT? OR PA=PVA OR PA=PATENTVERW? OR PA=GESELLSCHAFT OR PA=HANDELSKA? OR PA=INNOVATIONS-MAN? OR PA=INNOVECTIS OR PA=INNOWI OR PA=IPAL OR PA=SCIENCEBR? OR PA=MBM OR PA=VERWERT? OR PA=PATON OR PA=PROVENDIS OR PA=STEINBEIS OR PA=TECHNOLOGIE-LIZ? OR PA=TRANSMIT OR PA=FORSCHUNGF? OR PA=TUTECH OR PA=WIRTSCHAFTSF? OR PA=ZAB OR PA=ZUKUNFTSAG? OR PA=ZENTRALE)*

where (year) was replaced by the four-digit year without parentheses.
