# Supplementary material for: Patenting of University and Non-University Public Research Organisations in Germany: Evidence from Patent Applications for Medical Research Results
Source: PLoS One. 2010 Nov 18;5(11):e14059. doi: 10.1371/journal.pone.0014059 (PMC2987808; doi:10.1371/journal.pone.0014059)
Supplement: Annex S2 — Patent family requests. (0.03 MB DOC) [file pone.0014059.s002.doc]

# Annex S2 - Patent family requests

We used the DEPATISnet patent family search interface, available at:

<http://depatisnet.dpma.de/DepatisNet/depatisnet?action=familie>

Families were requested for each document giving the publication number as search criteria.
