# Supplementary material for: Patenting of University and Non-University Public Research Organisations in Germany: Evidence from Patent Applications for Medical Research Results
Source: PLoS One. 2010 Nov 18;5(11):e14059. doi: 10.1371/journal.pone.0014059 (PMC2987808; doi:10.1371/journal.pone.0014059)
Supplement: Annex S3 — SQL requests. (0.04 MB DOC) [file pone.0014059.s003.doc]

# Annex S3: SQL requests

## UNION requests

In steps Error: Reference source not found and Error: Reference source not found we combined information from the document table (TableError: Reference source not found respectively TableError: Reference source not found) and the applicant table (TableError: Reference source not found). We created new tables containing multiple entries for documents with multiple applicants, one entry for each applicant.

This was done automatically using the following SQL-request in Microsoft Access 2003 (step 10, for step 12 TableError: Reference source not found was replaced with TableError: Reference source not found):

*SELECT TableError: Reference source not found.*, TableError: Reference source not found.*
FROM TableError: Reference source not found INNER JOIN TableError: Reference source not found ON TableError: Reference source not found.Applicant = TableError: Reference source not found.Applicant1*

*UNION
SELECT TableError: Reference source not found.*, TableError: Reference source not found.*
FROM TableError: Reference source not found INNER JOIN TableError: Reference source not found ON TableError: Reference source not found.Applicant = TableError: Reference source not found.Applicant2*

*UNION
SELECT TableError: Reference source not found.*, TableError: Reference source not found.*
FROM TableError: Reference source not found INNER JOIN TableError: Reference source not found ON TableError: Reference source not found.Applicant = TableError: Reference source not found.Applicant3*

*UNION
SELECT TableError: Reference source not found.*, TableError: Reference source not found.*
FROM TableError: Reference source not found INNER JOIN TableError: Reference source not found ON TableError: Reference source not found.Applicant = TableError: Reference source not found.Applicant4*

*UNION
SELECT TableError: Reference source not found.*, TableError: Reference source not found.*
FROM TableError: Reference source not found INNER JOIN TableError: Reference source not found ON TableError: Reference source not found.Applicant = TableError: Reference source not found.Applicant5;*

## Selecting only university-related documents

In step Error: Reference source not found we selected only documents where at least one applicant is a university or university affiliated institution. This was done automatically using the following SQL-request in Microsoft Access 2003:

*SELECT TableError: Reference source not found.*
FROM TableError: Reference source not found WHERE
(TableError: Reference source not found.Applicant1Group="Error: Reference source not found" Or TableError: Reference source not found. Applicant2Group=" Error: Reference source not found" Or TableError: Reference source not found. Applicant3Group=" Error: Reference source not found" Or TableError: Reference source not found. Applicant4Group=" Error: Reference source not found" Or TableError: Reference source not found. Applicant5Group=" Error: Reference source not found");*
